# Dihydrotanshinone l alleviates psoriasis-like skin lesion *via* suppressing STAT3 signaling and DCs-Th17 responses

**DOI:** 10.1039/d6ra03228a

**Published:** 2026-07-22

**Authors:** Luoming Zhang, Xiaoyue Qi, Baodong Ma, Ranran Jin, Yiming Shao, Yaoxin Gao

**Affiliations:** a Cell Research and Transformation Center, Zhengzhou Central Hospital Affiliated to Zhengzhou University 16th, Tongbai North Road Zhengzhou 450007 China yaoxingao@zzu.edu.cn; b School of Life Sciences, Zhengzhou University Zhengzhou 450052 China

## Abstract

Skin psoriasis involves the activation of skin dendritic cells (DCs) by external triggers. Activated DCs then secrete interleukin 23 (IL-23), which acts as a connector between innate and adaptive immunity and promotes the differentiation of T helper 17 (Th17) cells. This process leads to an elevated production of interleukin 17 (IL-17) and excessive keratinocyte production, creating an autoimmune loop in the development of psoriasis. Dihydrotanshinone 1 (DIH), a flavonoid recognized for its antitumor effects, was assessed for its impact on imiquimod (IMQ)-induced psoriatic plaques in mice. In this investigation, our team used IMQ-induced mouse models to study how DIH affects keratinocytes and skin immunity. At first, we noticed that psoriasis-like lesions were significantly more severe in mice treated with DIH. However, after giving DIH to IMQ-induced mice, we observed a reduction in hyperproliferative keratinocytes, cytokine release, and the accumulation of DCs and Th17 cells. Further exploration showed that DIH relieved psoriasis-like skin conditions and inhibited the abnormal proliferation of HaCaT cells and Th17 cell differentiation. In particular, we found that DIH achieved these outcomes by modulating the STAT3 signaling pathway, which in turn suppressed DCs/Th17 cell responses and keratinocyte proliferation. Hence, DIH may affect the recruitment and function of immune cells in the skin and alter keratinocyte proliferation and differentiation in the context of psoriasis.

## Abstract 2

Dihydrotanshinone I alleviates psoriasis lesions *via* blocking STAT3 pathway and restraining DCs-Th17 mediated inflammatory responses. Image created with the assistance of Biorender AI.

## Graphical abstract

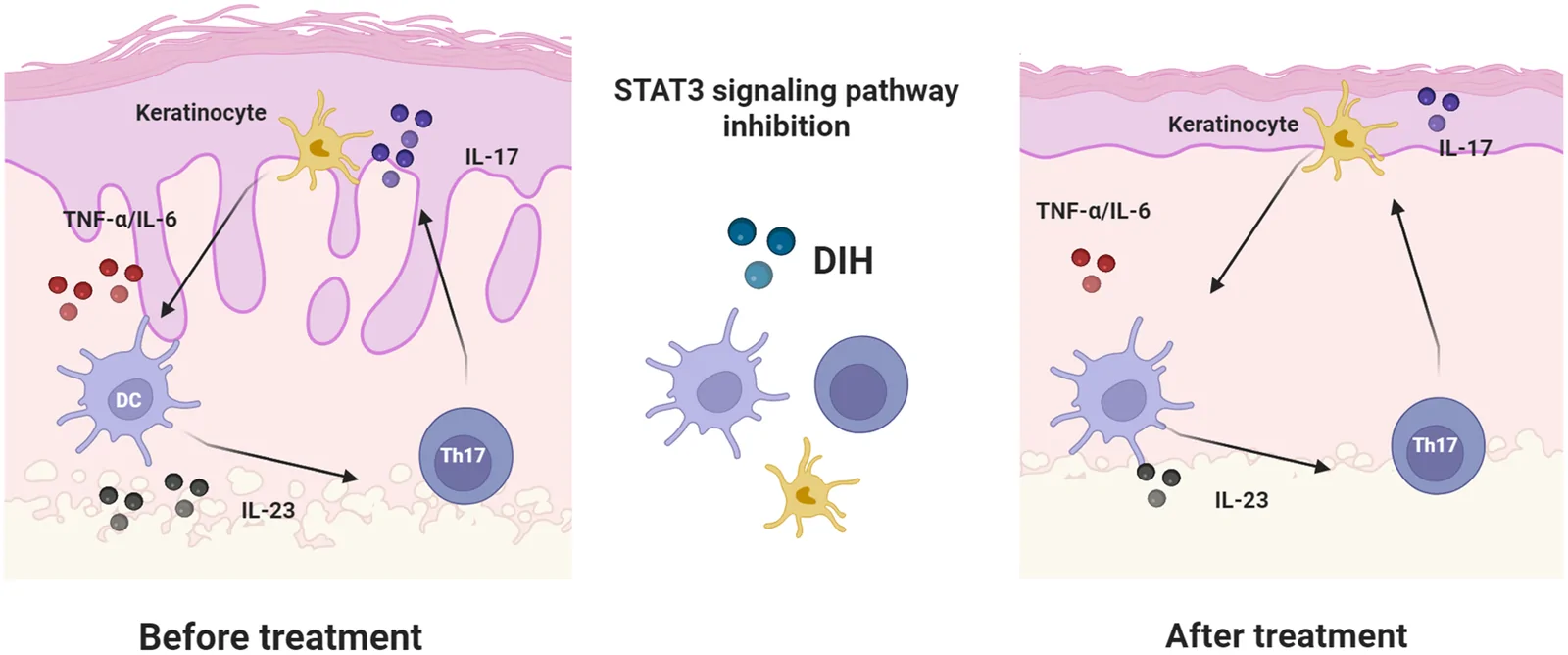

## 1 Introduction

Clinically, psoriasis is recognized by the triad of pruritus, well-demarcated erythematous plaques and silvery scale.^1,2^ A multitude of studies have shown that inflammatory infiltration in the skin is mainly initiated by various immune cells, including macrophages, DCs, lymphocytes, and neutrophils.^3,4^ These immune cells ultimately lead to the diverse proliferation and a low level of differentiation of keratinocytes.^5,6^ Additionally, the IL-23/IL-17 axis is deemed to hold vital importance in autoimmune inflammatory skin diseases. This axis operates through a mechanism in which activated dendritic cells (DCs), when exposed to unidentified external stimuli, control Th17 cells by secreting IL-23.^7^ Moreover, Th17 cells are capable of releasing cytokines like IFN-γ, TNF-α, and IL-17, which subsequently promote the proliferation of keratinocytes.^8^ This promotion serves as a significant contributor to the development of psoriatic lesions, leading to an enhanced production of growth factors and inflammatory cytokines in psoriasis.^9^

Psoriasis affects roughly 60 million people worldwide, with prevalence spanning 0.14–1.99%; East Asia reports the lowest rate at 0.14%.^10,11^ Current therapies, including topical agents, often carry adverse effects, underscoring the urgent need for safer alternatives. Topical corticosteroids cause skin atrophy, telangiectasia and rebound inflammation. Vitamin D analogues lead to local skin irritation and hypercalcemia. Oral methotrexate and cyclosporine induce organ toxicity, while IL-17/IL-23 biologics increase infection risk and have high medical costs.^12–16^ Traditional Chinese medicine (TCM), guided by holistic and syndrome-differentiated principles, has emerged as a clinically relevant option for preventing and managing psoriasis.^12,13^ Dihydrotanshinone 1 (DIH) is a lipophilic natural product isolated from the root of *Salvia miltiorrhiza*. Accumulating evidence shows that DIH exerts potent anti-tumor activity against liver, breast and colorectal cancers *via* targeting PKM2, ESR1, PGAM1 and KEAP1 in both cell-based assays and animal models.^17–23^

STAT3 is a central signaling molecule in psoriasis pathogenesis, playing critical roles in DCs, Th17 cells, and keratinocytes (KCs).^24,25^ In DCs, IL-23 activates STAT3 *via* JAK2/TYK2, promoting Th17 cell differentiation. Th17-derived cytokines such as IL-17A and IL-22 further stimulate KC proliferation, inhibit differentiation, and induce pro-inflammatory genes through the STAT3 pathway, forming a “feed-forward” inflammatory loop.^26–29^ Sustained STAT3 activation in KCs alone can spontaneously generate psoriatic lesions, while KC-specific STAT3 knockout significantly attenuates inflammation.^30^ Recent studies have further revealed that the p38α-STAT3 axis regulates KC proliferation, and the S1PR3-Src-STAT3 positive feedback loop maintains KC hyperproliferation and inflammatory states.^29,31^ Additionally, IL-6/STAT3 signaling contributes to neutrophil production, exacerbating lesional inflammation.^32^ JAK inhibitors targeting the STAT3 pathway have demonstrated clinical efficacy.^33^

In this research, we aimed to delve into the function and mechanism of how DIH impacts the autoimmune loop in psoriasis. The outcomes of our study indicated that DIH relieved psoriasis lesions in mice. This relief was closely related to a drop in the number of DCs and Th17 cells that congregated in the skin, which in turn caused a reduction in the secretion of inflammatory cytokines. These cytokines are the ultimate culprits behind epidermal hyperplasia. Consequently, a lower release of chemokines would hinder the recruitment of abnormal immune cells. Specifically, DIH made DCs secrete less IL-23, which suppressed the differentiation of Th17 cells and led to a decreased production of IL-17A. Furthermore, previous studies have demonstrated that DIH acts as a STAT3-targeted agent.^34–37^ Consistently, our findings showed that DIH disrupts the STAT3 signaling cascade to alleviate the development of psoriasis. In summary, these results underscore the therapeutic potential of DIH for the clinical treatment of psoriasis.

## 2 Methods

### 2.1 Exploring the promising targets of DIH in psoriasis

The GeneCards database served to identify potential therapeutic targets linked to psoriasis. These targets were submitted to the UniProt database (https://www.uniprot.org), and their visualization was realized through Venn analysis. For the construction of a protein–protein interaction (PPI) network, overlapping targets between DIH and the disease were uploaded to the STRING database (https://cn.string-db.org/). These targets were further loaded into Cytoscape 3.9.1, and core targets were determined on the basis of specific degree values. The main components were sourced from the RCSB PDB (https://www.rcsb.org/) and PubChem databases. After adding hydrogen atoms to receptor proteins and allocating charges, AutoDockTools 1.5.6 was used to create docking grid boxes at the active sites. In the final step, data visualization was achieved through PyMOL and LigPlot + software.^38^

### 2.2 Cell culture and viability

The HaCaT cell line, obtained from the Type Culture Collection Committee of the Chinese Academy of Sciences in Shanghai, China, was cultivated in DMEM medium supplemented with 10% FBS and 1% P/S (Cat. No. A5670701 and 15140122, Gibco, USA). Incubation of the cultures occurred at 37 °C in a 5% CO_2_ atmosphere. The cells were treated with 0, 5, 10, and 20 µM concentrations, or M5 (10 ng mL^−1^), for 24 and 48 hours. Cell viability was assessed *via* CCK8 (Cat. No. HY-K0301, MCE, USA) according to the manufacturer's instructions.

### 2.3 Animals

We obtained six- to eight-week-old C57BL/6 mice from Beijing Vital River Laboratory Animal Technology Co., Ltd (Beijing, China).^39^ These mice were randomly divided into four experimental groups. The allocation of each mouse to either the drug treatment group or the control group was determined by sealed envelopes that contained a random sequence generated using Excel software, with each group having 5 mice. With the exception of the control group (CON), every mouse was topically treated with 62.5 mg IMQ on the dorsal skin once daily for six days in a row.^40^ For *in vivo* use, DIH was dissolved in a minimal volume of DMSO and diluted with 0.5% CMC-Na sterile saline to obtain homogeneous suspension. The final content of DMSO was kept under 1%. Meanwhile, control group was established, and mice were given equal-volume blank solvent *via* intraperitoneal injection under the same intervention conditions. In the treatment group, low-dose DIH (Cat. No. HY-N0360, MCE, USA) (DIH- L) group (10 mg kg^−1^ intraperitoneal DIH injection); high-dose DIH (DIH-H) group (20 mg kg^−1^ intraperitoneal DIH injection);^41,42^ and methotrexate (MTX, 1 mg kg^−1^ orally administered, HY-14519, MCE) as a positive control group.^43^ A blinded 0–4 PASI system was applied. Upon reaching endpoints, euthanasia was performed with CO_2_.^43^ All animal experiments were conducted from May 1, 2025 to May 9, 2025 (8 days duration). The study protocol was reviewed and approved by the Institutional Animal Care and Use Committee (IACUC) of Zhengzhou University on April 1, 2025 (Approval No. 2025-YYY-012).

### 2.4 Skin histopathological examination

Tissue samples taken from the dorsal skin were fixed using a 4% paraformaldehyde (PFA) solution.^39^ After that, staining was carried out using the hematoxylin and eosin (HE) staining method. In immunohistochemical investigations, the primary antibodies used were rabbit anti-Ki67 (Catalog No. ab15580, Abcam, UK), PCNA (Catalog No. #13110, CST, USA), and K17 (Catalog No. #4543, CST, USA). On the other hand, the secondary antibody used was polymer-horseradish peroxidase-labeled goat anti-rabbit IgG (Catalog No. ab150077, Abcam, UK).

### 2.5 Immunofluorescence staining

The skin tissue specimens went through a series of procedures including paraffin embedding and tissue sectioning, along with other related steps.^39^ The resulting skin tissue sections were subjected to an overnight incubation with antibodies specific for CD4 (Cat. No. GB15064, Servicebio, China), IL-17 (GB11110, Servicebio, China), CD11C (GB155690, Servicebio, China), and IFN-γ (GB11107, Servicebio, China). Following the overnight incubation, appropriate secondary antibodies were applied to identify and visualize the expression levels of the target proteins.

### 2.6 qRT-PCR

Samples were processed to isolate total RNA *via* TRIzol reagent (Cat. No. 15596018CN, Invitrogen, USA). Then, cDNA synthesis was conducted with the PrimeScript™ RT Kit (Takara, Japan). Subsequently, the qPCR reaction was carried out on the Applied Biosystems 7500 Real-Time PCR System, with strict adherence to the manufacturer's protocols. The primers employed in the qRT-PCR assay are presented in Table S1.

### 2.7 ELISA

The inflammatory factors present in supernatant and the back skin extracts were quantified using ELISA kits obtained from Thermo Fisher Scientific, USA.^39^ Specifically, the levels of mouse IL-1β (88-7013A-88, Thermo Fisher Scientific, USA), mouse IL-17A (88-7371-88, Thermo Fisher Scientific, USA), mouse IL-23 (BMS6017, Thermo Fisher Scientific, USA), mouse TNF-α (BMS607-3, Thermo Fisher Scientific, USA), human IL-6 (88-7066-88, Thermo Fisher Scientific, USA), human TNF-α (D711045-0096, Sangon Biotech, China), human IL-1β (SEKH-0002, Solarbio, China), and IL-23 (BMS2013, Thermo Fisher Scientific, USA) were measured strictly in accordance with the manufacturer's provided instructions on a microplate reader (SpectraMax M2, Molecular Devices, USA).

### 2.8 Isolation of CD4^+^ T cells

We obtained naive CD4^+^ T cells from the splenocytes of C57BL/6 mice. To stimulate these cells, we used anti-CD3 mAb with a concentration of 1 µg mL^−1^ and anti-CD28 mAb at the same concentration of 1 µg mL^−1^. After that, the cells were placed in an incubation environment with IL-6 at 30 ng mL^−1^, TGF-β at 3 ng mL^−1^, IL-23 at 10 ng mL^−1^, anti-IL-17A mAb at 10 µg mL^−1^, and anti-IL-4 mAb at 10 µg mL^−1^, respectively. Three days into the incubation, we added PMA/ionomycin to the culture, along with GolgiPlug (BD Biosciences).^44^

### 2.9 Preparation of bone marrow-derived DCs

Bone marrow cells were harvested and then cultured in a medium composed of 10% fetal bovine serum (FBS), 100 units per milliliter (U mL^−1^) of penicillin–streptomycin (P/S), 10 nanograms per milliliter (ng mL^−1^) of granulocyte-macrophage colony-stimulating factor (GMCSF), and 5 ng mL^−1^ of interleukin-4 (IL-4) (all from Peprotech, USA). After three days, the supernatants in the culture plate were removed and replaced with fresh medium. On the fifth day, half of the medium in the plate was substituted with fresh medium.^44^

### 2.10 Flow cytometry

Spleen single-cell suspensions were prepared based on procedures detailed in earlier research.^7^ To start, the cells were stained with a range of cell-surface markers: Alexa Fluor 700 anti-CD45 mAb (147715, BioLegend, USA), FITC anti-CD3 mAb (100204, BioLegend, USA), and APC anti-CD4 mAb (116013, BioLegend, USA), APC anti-CD11c mAb (BioLegend, 117305, USA), PE anti-CD40 mAb (BioLegend, 124609, USA). For intracellular staining with PE-CY7 anti-IL-17 mAb (506921, BioLegend, USA) and PE anti-IFN-γ mAb (163503, BioLegend, USA), the cells were treated with phorbol 12-myristate 13-acetate/ionomycin for 4 hours, with GolgiPlug (BD Biosciences) added to the mixture. The samples were subsequently analyzed on an LSRFortessa flow cytometer (BD Biosciences) using FlowJo software (TreeStar).

### 2.11 RNA sequencing

Skin tissue samples underwent lysis with TRIzol before being sent to Sangon Biotech Co., Ltd (Shanghai, China) for RNA-seq analysis.^45^ Quantitative evaluation of gene expression levels was performed using RSEM software, and differentially expressed genes (DEGs) were identified *via* DESeq2 software. Various comprehensive analyses were performed, including we conducted Gene Ontology (GO) analysis, Kyoto Encyclopedia of Genes and Genomes (KEGG) pathway analysis, and target gene cluster analysis. The raw sequence data presented in this paper have been submitted to the Genome Sequence Archive (with the accession number GSA: CRA028115).

### 2.12 Cellular thermal shift assay (CETSA)

Lysates from HaCaT cells, prepared using M-PER reagent (78501, Thermo Fisher, USA) supplemented with protease and phosphatase inhibitors, were treated with either 20 µM of DIH or a DMSO vehicle control. After a 1 hour incubation at room temperature, the lysates were homogenized, aliquoted, and exposed to a temperature gradient in a thermocycler for 3 minutes. Following heat treatment, the samples were centrifuged at 20 000×*g* for 20 min to pellet denatured proteins. The resultant supernatants, representing the soluble protein fraction, were collected for subsequent immunoblotting analysis.

### 2.13 Western blotting

Protein samples were extracted from skin tissue using RIPA lysis buffer, followed by centrifugation at 12 000 rpm for 10 minutes. After quantitative denaturation, equal amounts of protein samples were separated using 10% SDS protein gels. Following the separation, the gels were transferred onto PVDF membranes. Initially, the membranes were incubated in 5% non-fat skim milk. Subsequently, they were incubated overnight at 4 °C with primary antibodies. The primary antibodies employed included Phospho-STAT3 (Tyr705) (Catalog No. ab308386, Abcam, USA), STAT3 (Catalog No. ab68153, Abcam, USA), and Actin (Catalog No. ab8227, Abcam, USA). After this procedure, the membranes were separately incubated with the corresponding secondary antibodies at room temperature.

### 2.14 Downregulation of STAT3

The interference sequences targeting STAT3 were obtained using an online shRNA design tool (https://rnaidesigner.thermofisher.com/rnaiexpress/sort.do), and these sequences are detailed in Table S2. The siRNAs were synthesized by Sangon Biotech Co., Ltd (Shanghai, China).^39^ Then, both the shRNA and the empty vector were introduced into HEK293T cells to package the lentivirus. Following this, the packaged lentivirus was co-cultured with the target cells for 48 hours. Ultimately, the cells were collected for further analysis.

### 2.15 Drug safety analyses

To determine the toxicity of DIH *in vivo*, the body weight and main organs of mice were monitored. The serum level of blood urea nitrogen (BUN), alanine aminotransferase (ALT) and alkaline phosphatase (ALP) were detected using BUN detection kit (Cat. No. abs580197, Absin), ALT detection kit (Cat. No. abs580002, Absin) and ALP detection kit (Cat. No. abs5520071, Absin), which were stored strictly according to the manufacturer's instructions to ensure their activity and stability.

### 2.16 Statistical analysis

Analysis of the complete dataset was performed with GraphPad Prism 6.0 software. Statistical evaluations depended on the one-way ANOVA test, and findings were reported as mean ± SD. A *p*-value below 0.05 was used to define a significant difference. Across all experimental designs, each experimental condition comprised no fewer than three replicate experiments.

## 3 Results

### 3.1 DIH inhibited abnormal proliferation and inflammatory response in M5-induced HaCaT cells

**Fig. 1:**
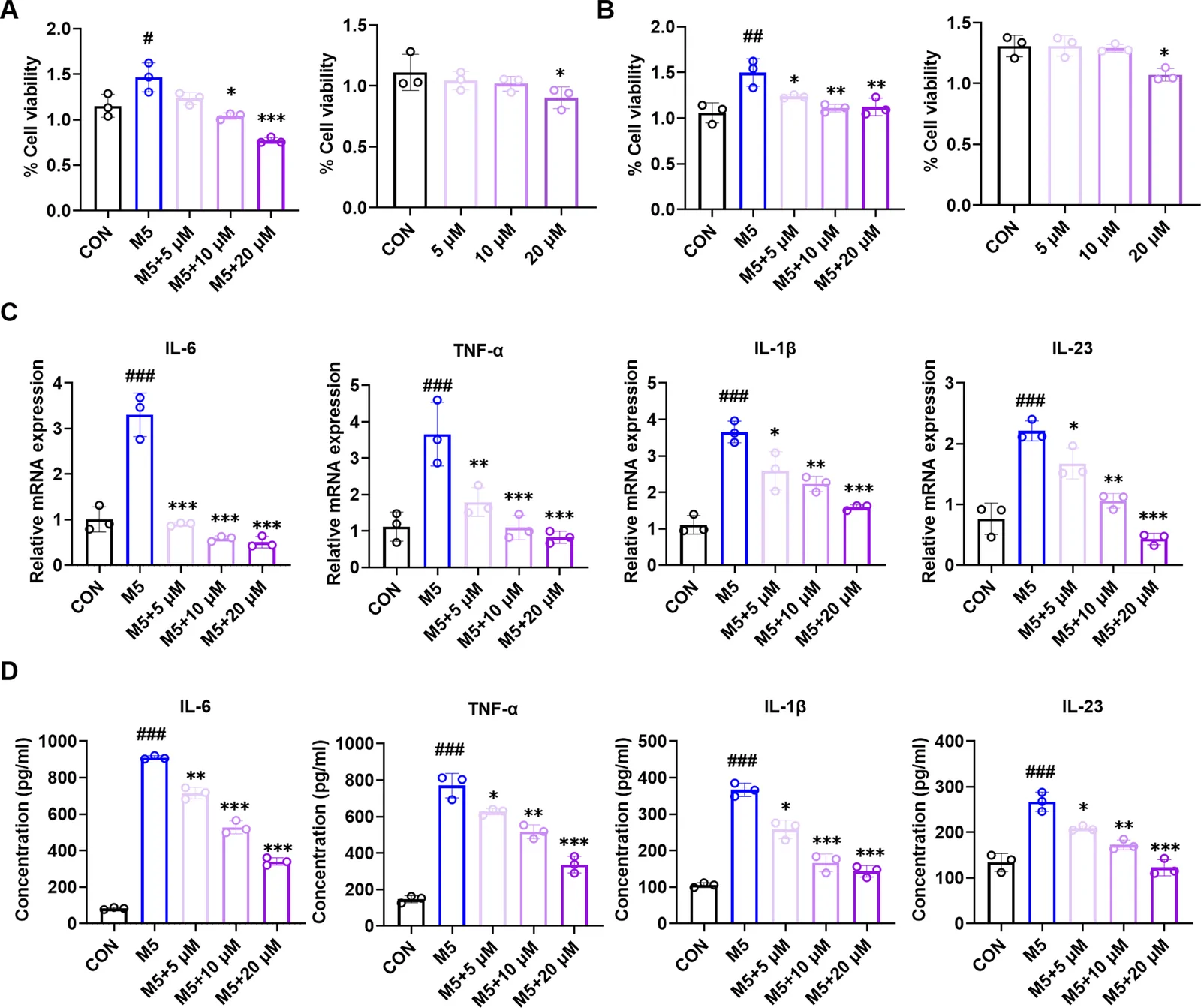
The ramifications of DIH concerning cell proliferation and inflammatory responses. (A and B) The CCK-8 was utilized to assess the impact of DIH at concentrations of 0, 5, 10, and 20 µM on the viability of HaCaT cells following 24 h and 48 h incubation periods. Additionally, the CCK-8 was applied to measure the viability of HaCaT cells that had been exposed to M5 (10 ng mL^−1^) and different concentrations of DIH (ranging from 0 to 20 µM) for 24 h and 48 h. (C and D) For the quantification of the mRNA and protein expression levels of IL-6, TNF-α, IL-1β, and IL-23 in HaCaT cells, qRT-PCR and ELISA were carried out after the cells were treated with M5 (10 ng mL^−1^) and DIH (0-20 µM) for a duration of 12 h and 16 h, separately. All experiments were repeated in triplicate. *P* values were calculated using one-way ANOVA followed by Turkey's test. (*n* = 3, Mean ± SD; *vs.* CON, ^#^*p* < 0.05, ^##^*p* < 0.01, ^###^*p* < 0.001; *vs.* M5, *p < 0.05, **p < 0.01, ***p < 0.001).

HaCaT cells were employed to assess the impact of DIH on cell proliferation and the inflammatory response. These HaCaT cells were treated with DIH at concentrations of 0, 5, 10, and 20 µM, either in the presence or absence of M5, for durations of 24 h and 48 h. Based on the CCK-8 assay results, the cell viability of the DIH-treated groups was similar to that of the control group (CON). Significantly, DIH effectively suppressed the abnormal proliferation of keratinocytes induced by M5, showing a dose-dependent inhibitory pattern (Fig. 1A and B). At the mRNA level, M5 markedly upregulated the expression of inflammatory cytokines IL-6, TNF-α, IL-1β, and IL-23, reaching 2.2–4.0-fold of the control group (all *p* < 0.001). Consistently, their protein concentrations in the culture supernatant also increased to 2.3–9.0-fold of baseline levels (all *p* < 0.001). DIH co-treatment suppressed these elevations in a concentration-dependent manner. It reduced cytokine mRNA expression to 0.3–0.6-fold and protein levels to 0.3–0.4-fold at 20 µM compared to M5 group (all *p* < 0.001) (Fig. 1C and D). These observations indicated that DIH inhibited the proliferation of M5-stimulated HaCaT cells, offering a promising therapeutic option to combat excessive cell proliferation and the release of pro-inflammatory cytokines.

### 3.2 DIH alleviates the skin damage caused by IMQ

**Fig. 2:**
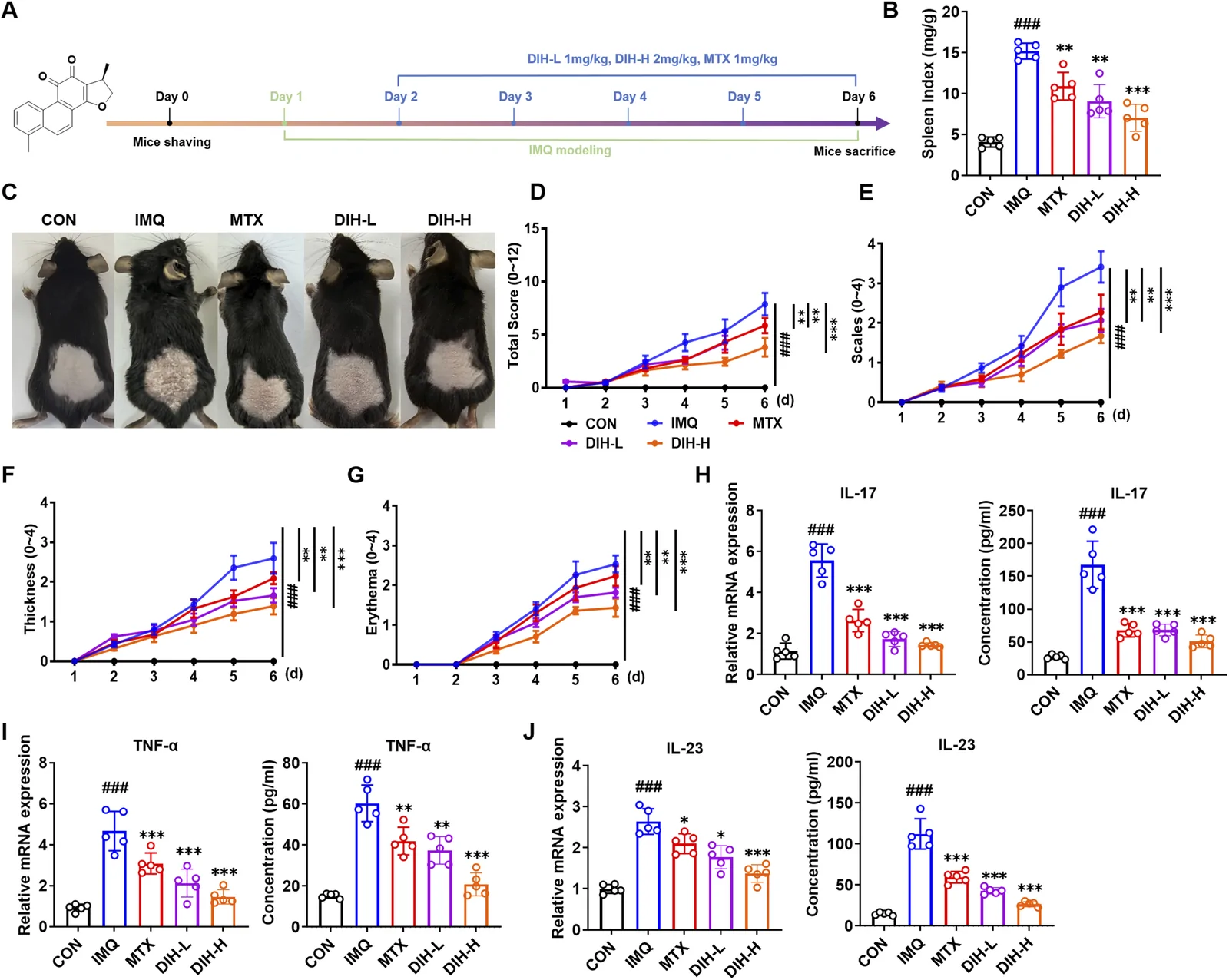
DIH improved psoriatic skin lesions in IMQ-triggered mice. (A) Experimental protocol: following dorsal hair shaving, mice (except for the control group) were given 62.5 mg of IMQ daily over the period from day 1 to day 6 (*n* = 5). (B) The splenic index was worked out for all groups. (C) Representative photographs of mouse skin lesions on Day 6. (D) The PASI scores were determined. (E–G) Daily evaluations were performed to score the degree of scaling, epidermal thickening, and erythema. (H–J) qRT-PCR and ELISA were employed to evaluate the transcript and protein levels of inflammatory cytokines (IL-17, TNF-α, IL-1β, IL-23), and β-actin was used for normalization. *P* values were calculated using one-way ANOVA followed by Turkey's test. (*n* = 3, Mean ± SD; *vs.* CON, ^####^*p* < 0.0001; *vs.* IMQ, *p < 0.05, **p < 0.01, ***p < 0.001).

In order to investigate the protective effect against psoriasis, we established an IMQ-induced psoriasis-like lesion model (Fig. 2A). By the sixth day, treatment of DIH successfully returned the spleen size to normal and significantly lowered the spleen index (Fig. 2B). Moreover, Mice in the model group manifested the hallmark symptoms of psoriasis, such as skin becoming red, skin lesions getting thicker, and scaling. On the other hand, mice treated with a low dose of DIH (DIH-L) and a high dose of DIH (DIH-H) showed a considerable reduction in these symptoms (Fig. 2C). Despite being effective compared to the IMQ group, DIH outperformed the MTX groups in terms of therapeutic efficacy, with noticeable improvements in scale formation, a decrease in skin thickness, and a reduction in erythema (Fig. 2D–G). The results unequivocally demonstrated that DIH could relieve the symptoms of IMQ-induced psoriasis-like skin inflammation in mice.

The inflammatory response stands as one of the pivotal factors driving the pathogenesis of psoriasis. Hence, curbing cytokine production is also among the key strategies for mitigating psoriasis.^46,47^ To achieve a deeper and more mechanistic understanding of how DIH intervened in the pathogenic cascade of psoriasis, we carried out an exhaustive, multi-layered profiling of the key inflammatory cytokines that fueled disease maintenance and progression. IMQ markedly upregulated the mRNA expression of key psoriasis-related cytokines IL-17, TNF-α, and IL-23 to 5.6-fold, 4.5-fold, and 2.6-fold of control levels (all *p* < 0.001), with corresponding protein concentrations elevated to 6.8-fold, 4.3-fold, and 6.1-fold of baseline (all *p* < 0.001). Compared with the IMQ group, treatment with methotrexate (MTX), DIH-L, and DIH-H significantly suppressed these elevations: DIH-H reduced IL-17, TNF-α, and IL-23 mRNA levels to 1.4-fold, 1.2-fold, and 1.4-fold of control, respectively, while their protein concentrations were decreased to 1.9-fold, 1.3-fold, and 1.6-fold of baseline (all *p* < 0.001 *vs.* IMQ) (Fig. 2H–J). Taken together, these findings established that DIH exerted a potent, multi-node suppression of the IL-23/Th17 inflammatory circuitry, providing a clear mechanistic underpinning for its therapeutic efficacy in psoriatic lesions.

### 3.3 DIH restrains IMQ-induced keratinocyte proliferation

**Fig. 3:**
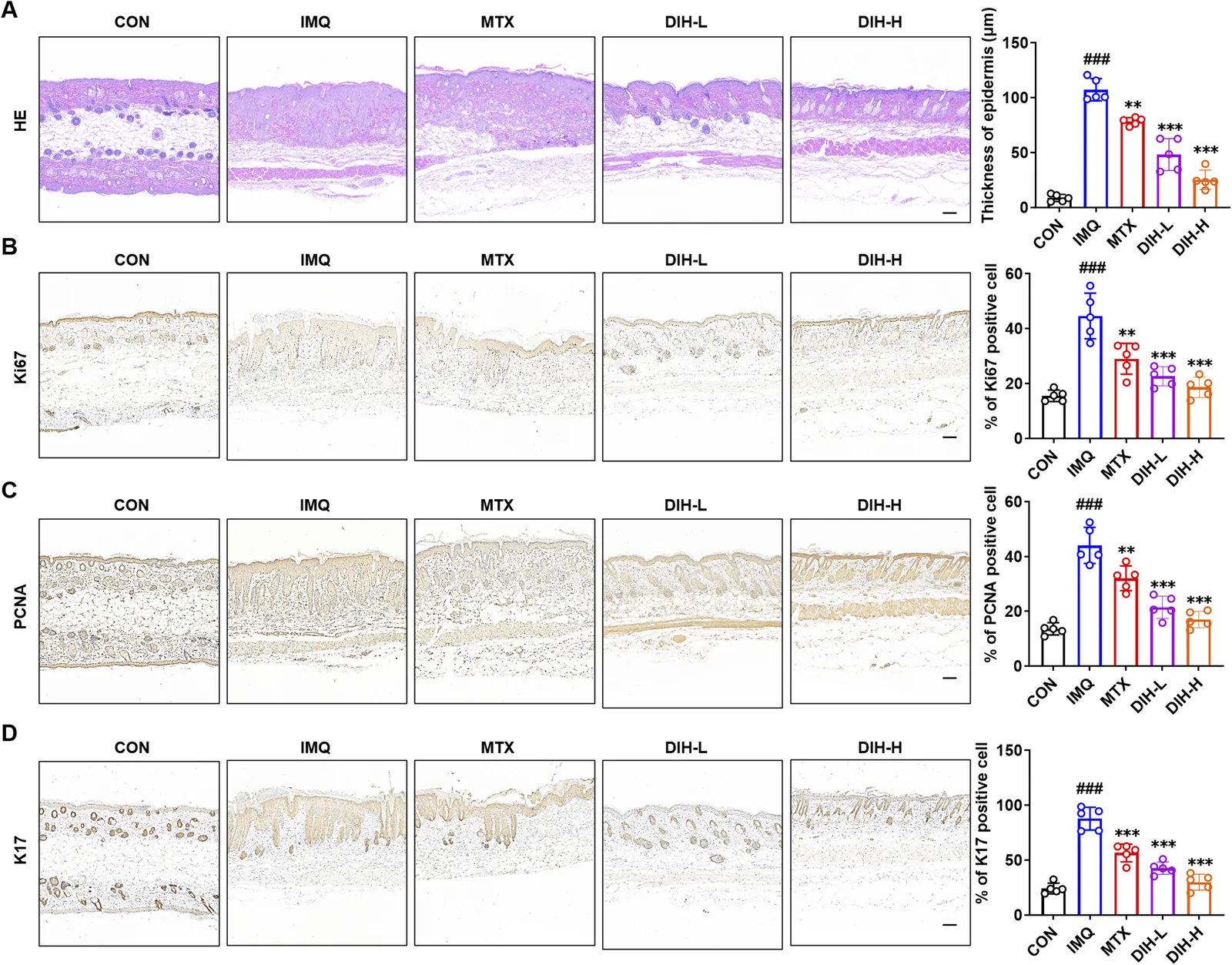
DIH relieved symptoms in IMQ-induced psoriatic mice. (A) We conducted Hematoxylin-eosin (HE) staining on psoriatic skin lesions and measured epidermal thickness quantitatively. (B) Ki67 immunostaining was done, and positive cells were counted. (C) PCNA immunostaining was performed, and the number of positive cells was determined. (D) K17 immunohistochemistry was carried out, and positive cells were counted (Bar = 100 µm). *P* values were calculated *via* one-way ANOVA and then Tukey's test. (*n* = 3, Mean ± SD; *vs.* CON, ^###^*p* < 0.001; *vs.* IMQ, **p < 0.01, ***p < 0.001).

Cytokine clustering interacts with immune cells, leading to the development of psoriasis.^48^ As depicted in Fig. 3A, there were a conspicuous decrease in epidermal thickening and infiltration of inflammation after the administration of DIH or MTX (Table S3). Compared with the CON group, IMQ group exhibited a robust in the epidermal basal and supranasal layers. However, mice receiving intraperitoneal injection of DIH displayed a sharp reversal of these indices. Consistent with these morphological changes, immunohistochemical staining for proliferation markers showed that IMQ significantly upregulated the percentages of Ki67-, PCNA-, and K17-positive cells, reaching ∼3.5-fold, ∼3-fold, and ∼4-fold of the control levels, respectively (all *p* < 0.001). Compared with the IMQ group, MTX and DIH treatments significantly reduced these proliferative indices. Notably, DIH-H reduced Ki67-, PCNA-, and K17-positive cell percentages to ∼1.3-fold, ∼1.5-fold, and ∼1.5-fold of the control, respectively, corresponding to 63%, 50%, and 63% reductions from the IMQ group (all *p* < 0.001) (Fig. 3B–D). Collectively, these data indicated that DIH not only halted cell-cycle re-entry and DNA synthesis but also disrupted the pathological keratinocyte differentiation program, suggesting that DIH treatment had the potential to reduce keratinocyte proliferation in psoriasis-afflicted mice.

### 3.4 DIH inhibits the differentiation of Th17 cells induced by BMDCs

**Fig. 4:**
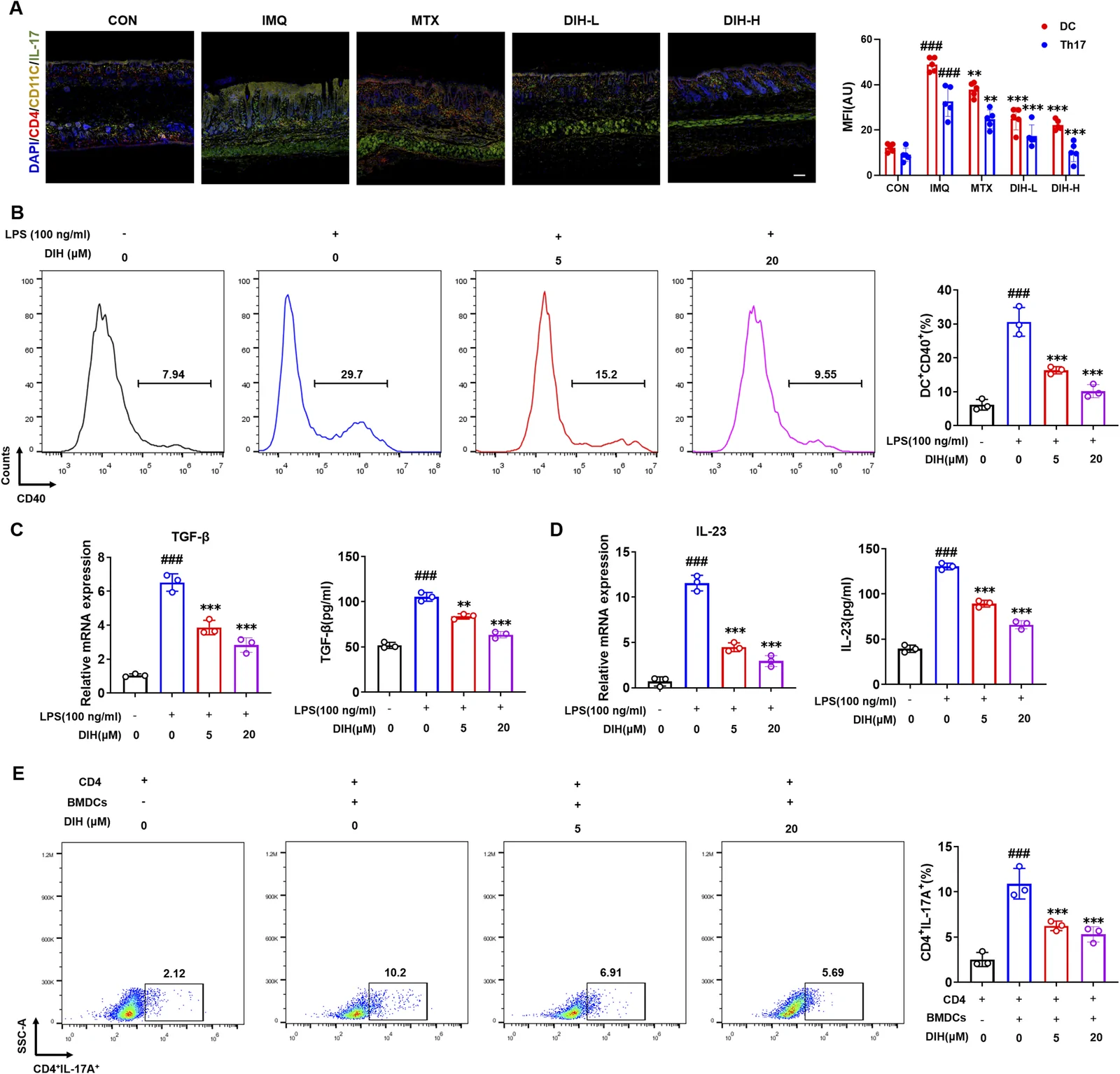
DIH markedly inhibited the activation process of DCs, which in turn modulated the differentiation of Th17 cells. (A) Immunofluorescence analysis of CD4, DC, and IL-17 co-localization in skin tissue (Bar = 100 µm). (B) BMDCs were isolated from spleen of mice. These cells were then stimulated with lipopolysaccharide (LPS) at a concentration of 100 ng mL^−1^, either in the presence or absence of DIH (at doses of 0 µM, 5 µM, and 20 µM) for a duration of 12 h. Subsequently, the expression of CD40 on BMDCs using FACS. (C and D) qRT-PCR and ELISA were employed to evaluate the transcript and protein levels of inflammatory cytokines (TGF-β and IL-23), and β-actin was used for normalization. (E) The differentiation of T cell subsets, which originated from naïve CD4^+^ T cells co-cultured with BMDCs that had been treated with DIH (at doses of 0 µM, 5 µM, and 20 µM) for 24 h, was evaluated by measuring the intracellular IL-17A. *P* values were calculated using one-way or two-way ANOVA followed by Turkey's test. (Mean ± SD; *vs.* CON, ###*p* < 0.001; *vs.* IMQ, **p < 0.01, ***p < 0.001).

The IL-17-secreting Th17 cells have downstream pro-inflammatory influences on the skin, giving rise to the pathophysiological traits and clinical symptoms.^49,50^ DCs are responsible for producing TGF-β and IL-23, which are involved in regulating the differentiation of Th17 cells.^51^ Our data distinctly revealed that DIH exerted a pronounced and statistically significant dampening effect on the pathogenic infiltration of both DCs and Th17 lymphocyte subsets within the lesional skin (Fig. 4A). With the aim of delving deeply into the precise mechanism of DIH's influence on skin DCs in the process of psoriasis development, we adopted murine BMDCs as a model to simulate skin DCs for *in vitro* investigations. We used FACS to evaluate the expression level of CD40 on DCs. The experimental data revealed that DIH significantly inhibited the maturation and production of TGF-β and IL-23 of BMDCs to reduce Th17 differentiation (Fig. 4B–E). This suggests that DIH suppresses Th17 cell differentiation to relieve skin lesions by regulating the cytokines secreted by DCs.

### 3.5 DIH improved psoriasis *via* STAT3 signaling pathway

**Fig. 5:**
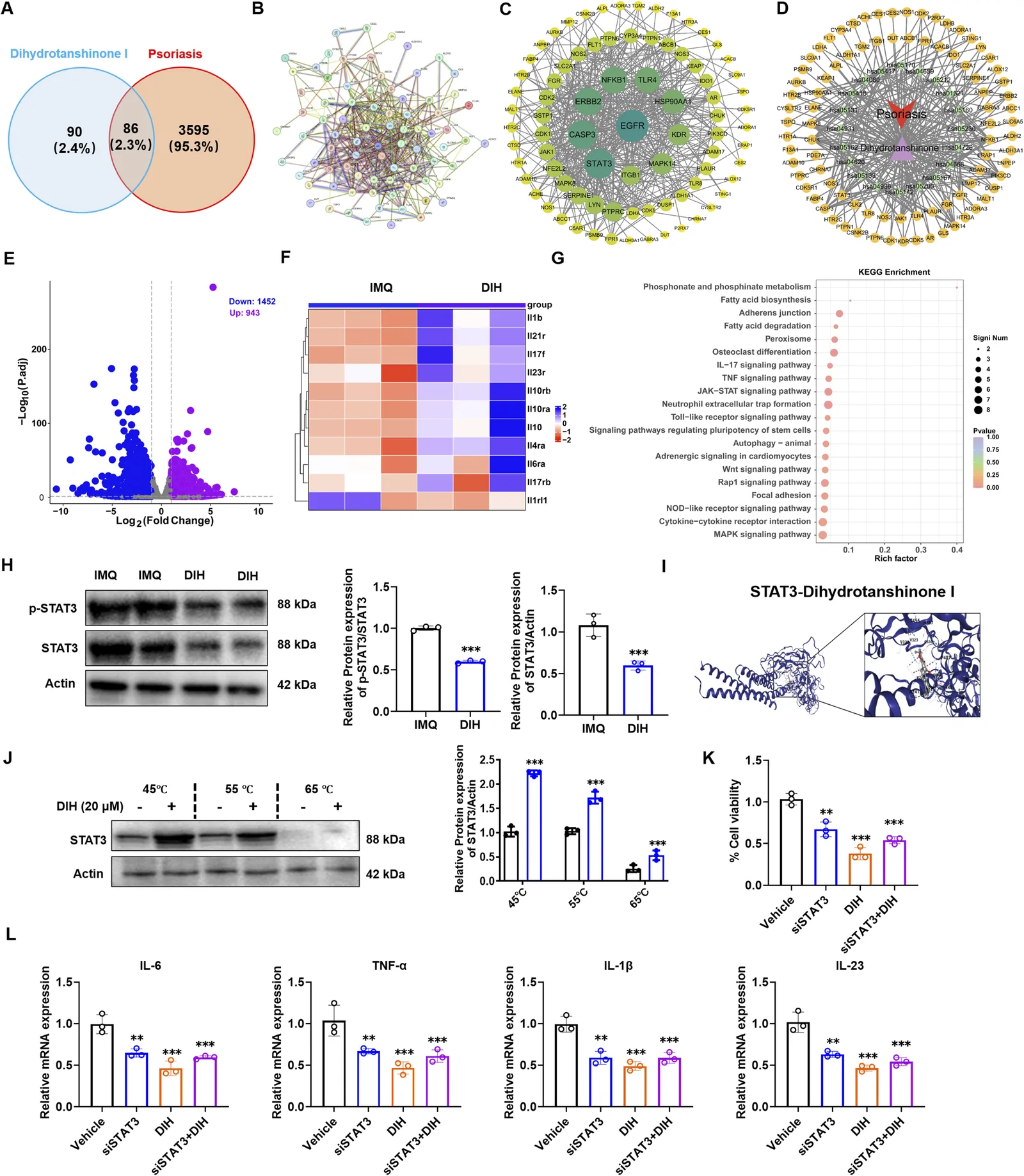
DIH inhibited skin lesions through the STAT3 signaling pathway. (A) We show a Venn diagram presenting the overlapping targets of DIH and psoriasis. (B and C) PPI networks are presented to reveal how DIH acts against psoriasis. (D) The diamond indicates the main signaling pathway related to psoriasis, and the circles show the common targets between DIH and psoriasis. (E) A volcano plot is used to display transcriptomic changes. (F) A heatmap is utilized to illustrate co-regulated target genes. (G) KEGG pathway analysis is done for gene functional annotation. (H) We confirm pathway suppression by measuring the phospho/total ratios of STAT3. Quantification of phospho/total ratios of STAT3 and STAT3/Actin ratios is shown in the bar graph. (I) Computational modeling is used to study the binding interactions between DIH and STAT3. (J) CETSA is performed for STAT3.Quantification of STAT3/Actin ratios is shown in the bar graph. (K and L) HaCaT cells are infected with sh-STAT3 lentivirus and treated with IL-17A (100 ng mL^−1^) or plus DIH (20 µM) for 24 h. CCK-8 and qRT-PCR are applied to assess cell proliferation and gene expression. *P* values were calculated using one-way or two-way ANOVA followed by Turkey's test. (Mean ± SD; *n* = 3; *vs.* Vehicle, **p < 0.01, ***p < 0.001).

To explore the potential targets of DIH and psoriasis, we screened many databases and obtained that 86 drug-disease intersecting targets (Fig. 5A). These candidate targets of DIH for psoriasis were then uploaded into the STRING database to show PPI network (Fig. 5B). Next, EGFR, STAT3, CASP3, NFKB1, TLR4, HSP90AA1, ERBB2, KDR, MAPK14 and ITGB1 were selected with the cytoHubba plugin (version 2.0) of Cytoscape 3.9.1 and located in the central region of the PPI network (Fig. 5C) (Table S4). Furthermore, the drug-target-pathway-disease network offered a straightforward visualization of the potential targets of DIH in psoriasis (Fig. 5D). To delve deeper into the mechanism underlying DIH's action in psoriasis, RNA sequencing (RNAseq) was carried out on skin tissues from the IMQ and DIH groups. As illustrated in Fig. 5E, a total of 2395 differentially expressed genes (DEGs) were detected in the mice of the DIH group, with 943 upregulated and 1452 downregulated. Additionally, enrichment analysis of the DEGs demonstrated that, in comparison to the IMQ group, the DIH group exhibited decreased expression of key genes (Fig. 5F). A subsequent Kyoto Encyclopedia of Genes and Genomes (KEGG) pathway analysis of these DEGs revealed a strong association between the JAK-STAT signaling pathway and the progression of IMQ-induced psoriasis (Fig. 5G). Next, we combined existing literature and sequencing results to detect the STAT3 signaling pathway.^52^ The data showed that DIH treatment significantly inhibited the phosphorylation of STAT3 (Fig. 5H). To examine interactions between DIH and key targets, molecular docking was conducted, showing that the binding energy of DIH with STAT3 was −6.7 kcal mol^−1^ (Fig. 5I). Moreover, DIH treatment caused STAT3 to be more stable to thermal destabilization compared to the DMSO control (Fig. 5J). Furthermore, our data showed that downregulation of STAT3 suppressed the IL-17A induced cell proliferation and cytokines production (Fig. 5K and L). These findings suggest that DIH alleviates psoriasis by diminishing the activity of the STAT3 pathways.

### 3.6 Safety of DIH administration

**Fig. 6:**
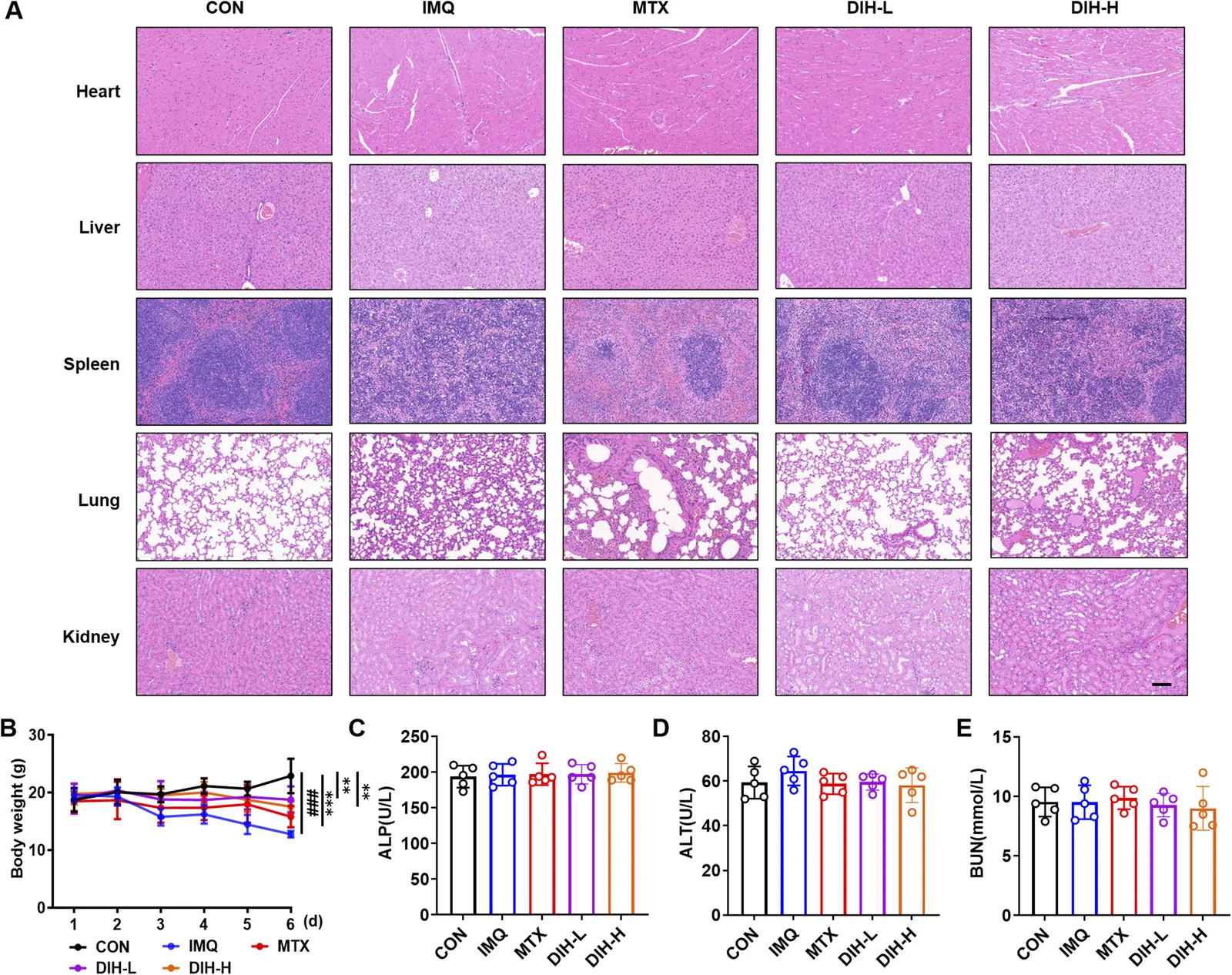
Safety of DIH administration. (A) At the experimental endpoint, heart, liver, spleen, lung, and kidney were collected and sections analyzed by HE staining (Bar = 100 µm). (B) Body weights of mice were measured at different time points. (C–E) Serum was collected and the concentration of defined serum factors determined. *P* values were calculated using one-way ANOVA followed by Turkey's test. (Mean ± SD; *vs.* CON, ###*p* < 0.001; *vs.* IMQ, **p < 0.01, ***p < 0.001).

In order to evaluate the possible dose toxicity of DIH, we first detected the change of organs in mice, and no obvious damage was displayed in all organs (Fig. 6A). Next, we kept a close eye on the body weight of all mice every day. From the very first day of feeding, the mice in the IMQ group saw a notable decrease in their body weight. But this decline was ameliorated when treated with MTX and DIH. Interestingly, the data showed that mice treated with DIH lost less weight than those treated with MTX (Fig. 6B). The renal and liver function indicators include ALP, ALT, BUN, *etc.* (Guo *et al.* 2024). Additionally, no obvious differences were found in the renal and liver functions across each group (Fig. 6C–E). We may thus draw the conclusion that dosing DIH at 10 mg kg^−1^ and 20 mg kg^−1^ is relatively safe in the context of mice.

## 4 Discussion

The defining features of psoriasis are persistent inflammatory responses and the secretion of diverse cytokines by immune cells, and these cytokines are the driving force behind the heightened proliferation of keratinocytes.^53^ The dysregulation of skin immune cells triggers inflammation, thereby facilitating the advancement of psoriatic lesions.^54^ Hyperproliferative keratinocytes are set off by cytokines secreted by immune cells, including TNF-α, IFN-γ, and IL-23/IL-17A.^55^ These cytokines prompt the generation of anti-microbial peptides, along with additional chemokines and cytokines. These substances not only drive the further hyperproliferation of keratinocytes but also attract immune cells such as Th17 cells, mDCs, neutrophils, and macrophages, leading to the establishment of an autoimmune amplification loop.^53,56^ In modern times, topical treatments come with significant adverse effects, underscoring the importance of investigating new therapeutic methods.^57^ Consequently, Traditional Chinese Medicine (TCM), which includes substances like benzoylaconitine and liquiritin, is receiving increasing attention as a remedy for psoriasis.^40,48^ DIH exhibits significant anti-inflammatory effects and regulates the function of immune cells, particularly inhibiting the abnormal activation of cells to balance immune responses.^58^ In terms of anti-proliferation, it can inhibit the growth and proliferation of tumor cells.^21,23^ Additionally, it possesses certain antioxidant properties and potential cardiovascular protective effects. These multi-target characteristics enable it to show potential therapeutic value in the fields of inflammatory diseases, autoimmune diseases, and tumors. The results reported in this study demonstrate that DIH reduced the psoriasis reaction through curbing abnormal cell proliferation, the synthesis of cytokines, and the movement of immune cells.

In mice subjected to the IMQ-only model, distinct psoriasis-like skin symptoms were observed. The histopathological features matched those of psoriasis, confirming that IMQ successfully induced a psoriasis-like disease model in this study, which is consistent with previously published research.^59,60^ It has been verified that daily topical application of DIH can alleviate skin symptoms and reduce skin lesions *in vivo*. At the peak of the drug-induced disease, the mice displayed an increase in spleen weight, substantial body weight loss, and a higher spleen index, which serves as a crucial parameter for evaluating the progression of psoriasis.^61^ In the present study, the model group demonstrated a notable rise in the spleen index. Nevertheless, after DIH treatment, the body weight of the mice rebounded, and spleen enlargement was significantly curbed, suggesting that DIH has ameliorated abnormal immune states.

The elevated expression of Ki-67 and proliferating cell nuclear antigen (PCNA) serves as a crucial diagnostic marker for hyperproliferation, differentiating psoriatic skin from normal skin. Elevated Ki-67 levels are a reflection of disease activity in psoriatic skin, while higher PCNA levels signify increased DNA synthesis activity.^62^ Keratin 17 (K17) plays a pivotal role in the pathogenesis of psoriasis. Its abnormal over-expression promotes excessive proliferation of keratinocytes (KCs), suppresses apoptosis, and acts as an autoantigen to activate T cells, especially Th17 cells, which then trigger the secretion of cytokines such as IL-17 and IL-23.^47,62^ This establishes a pro-inflammatory feedback loop, and the interaction between K17 and immune cells sustains chronic inflammation in psoriatic lesions, making K17 a potential therapeutic target for psoriasis.^5,63^ The findings of our study showed that DIH effectively downregulated the overexpression of Ki67, PCNA, and K17 in lesional skin.

Cytokines like IL-1β, TNF-α, IL-23, and IL-17 contribute to the accumulation of immune cells and boost keratinocyte proliferation. A previous study demonstrated that thalidomide could reduce IL-1β expression to suppress the inflammatory response in psoriasis.^9^ Dendritic cells expressing CD40/CD86 are capable of releasing pro-inflammatory cytokines, such as IL-6, to induce the differentiation of Th17 cells through pattern-recognition mechanisms.^64,65^ To gain insights into the function of DIH in influencing DCs in psoriasis, we established an IMQ-induced psoriasis mouse model for further evaluation. We observed that an accumulation of DCs and Th17 cells was also detected. Additionally, the upregulation of IL-6, TNF-α, IL-17A, and IL-23 was in line with previous findings. Furthermore, our research results demonstrated that DIH suppressed the differentiation of naïve CD4^+^ T cells into Th17 cells regulated by BMDCs, which supported the notion that DIH can mediate Th17 differentiation by regulating DCs. Our findings reveal that DIH has the ability to lower the expression of a variety of secreted cytokines and inhibit the role of DCs in controlling Th17 cell differentiation. As a result, it can alleviate the symptoms of psoriasis and aid in lessening the inflammatory conditions in skin lesions.

STAT3 is a key transcription factor that plays a central role in inflammatory responses. It can be activated by various pro-inflammatory cytokines, thereby regulating the expression of a large number of downstream inflammation-related genes, promoting the synthesis and release of pro-inflammatory factors, amplifying the inflammatory cascade, and maintaining a chronic inflammatory state.^66,67^ In psoriasis, the abnormal activation of STAT3 is an important mechanism underlying the occurrence and development of the disease: on the one hand, it can stimulate the abnormal proliferation of keratinocytes and inhibit their apoptosis, leading to excessive epidermal hyperplasia; on the other hand, its activation can promote the differentiation of Th17 cells and the secretion of cytokines such as IL-17, enhance immune cell infiltration and inflammatory responses, and form a vicious cycle of “inflammation-proliferation”.^68,69^ Therefore, STAT3 has become a highly promising target in the treatment of psoriasis.^70,71^ Molecular docking analysis revealed that DIH forms a stable complex with the coiled-coil domain (CCD) of STAT3, interacting with residues including V322, V323, Q326, T456, H457, and K244 *via* hydrogen bonds and hydrophobic interactions. This binding site is in close spatial proximity to the Tyr705 phosphorylation-containing transactivation domain of STAT3. The stable occupation of this region by DIH induces local steric hindrance and conformational perturbation, which blocks the recruitment and access of upstream kinases to the Tyr705 phosphorylation site. Consequently, STAT3 phosphorylation and subsequent dimerization are inhibited, leading to suppression of downstream signaling. These structural observations provide a direct molecular mechanism explaining the anti-psoriatic effect of DIH *via* STAT3 inhibition. Our research demonstrated that DIH suppressed STAT3 phosphorylation, and this suppressive effect further regulated both cell proliferation and the expression levels of inflammatory cytokines. However, in this study, there is a potential limitation that is the different administration routes used for DIH (intraperitoneal) and MTX (oral). While i.p. injection was chosen to ensure reliable systemic exposure of DIH, future studies evaluating oral formulations of DIH are warranted to better mimic clinical administration.

## 5 Conclusions

Our investigation revealed that DIH acts as a multi-target agent in combating psoriasis. It not only suppresses the excessive proliferation of keratinocytes but also re-establishes equilibrium in the abnormal DCs/Th17 immune responses through the precise modulation of the STAT3 axis. Hence, it stands out as a promising therapeutic candidate for the treatment of psoriasis.

## Author contributions

Luoming Zhang and Xiaoyue Qi: writing-original draft, validation, methodology, data curation, software. Luoming Zhang, Baodong Ma, Ranran Jin and Yiming Shao: software, methodology, formal analysis. Yaoxin Gao and Baodong Ma: visualization, funding acquisition. Yaoxin Gao: writing-review & editing, supervision, project administration.

## Conflicts of interest

All authors state that they have no conflicts of interest regarding the publication of this paper.

## Supplementary Material

RA-016-D6RA03228A-s001

RA-016-D6RA03228A-s002

RA-016-D6RA03228A-s003

RA-016-D6RA03228A-s004

RA-016-D6RA03228A-s005

## Data Availability

The data supporting the findings of this study are available from the corresponding author upon reasonable request. The raw sequence data presented in this paper have been submitted to the Genome Sequence Archive (with the accession number GSA: CRA028115). Supplementary information (SI) is available. See DOI: https://doi.org/10.1039/d6ra03228a.
