# Supplementary material for: Dihydrotanshinone l alleviates psoriasis-like skin lesion via suppressing STAT3 signaling and DCs-Th17 responses
Source: RSC Adv. 2026 Jul 22;16(38):42443–56. doi: 10.1039/d6ra03228a (PMC13390720; doi:10.1039/d6ra03228a)
Supplement: Supplementary file 4 [file RA-016-D6RA03228A-s004.pdf]

This journal is © The Royal Society of Chemistry 2026

**Table S4 The 10 most significant targets of PPI network.**

| Number | Target name | Degree |
|---|---|---|
| 1 | EGFR | 47 |
| 2 | STAT3 | 44 |
| 3 | CASP3 | 35 |
| 4 | NFKB1 | 34 |
| 5 | TLR4 | 31 |
| 6 | HSP90AA1 | 31 |
| 7 | ERBB2 | 30 |
| 8 | KDR | 26 |
| 9 | MAPK14 | 25 |
| 10 | ITGB1 | 22 |
