# Supplementary material for: Dihydrotanshinone l alleviates psoriasis-like skin lesion via suppressing STAT3 signaling and DCs-Th17 responses
Source: RSC Adv. 2026 Jul 22;16(38):42443–56. doi: 10.1039/d6ra03228a (PMC13390720; doi:10.1039/d6ra03228a)

Supplementary Information (SI) for RSC Advances.
This journal is © The Royal Society of Chemistry 2026

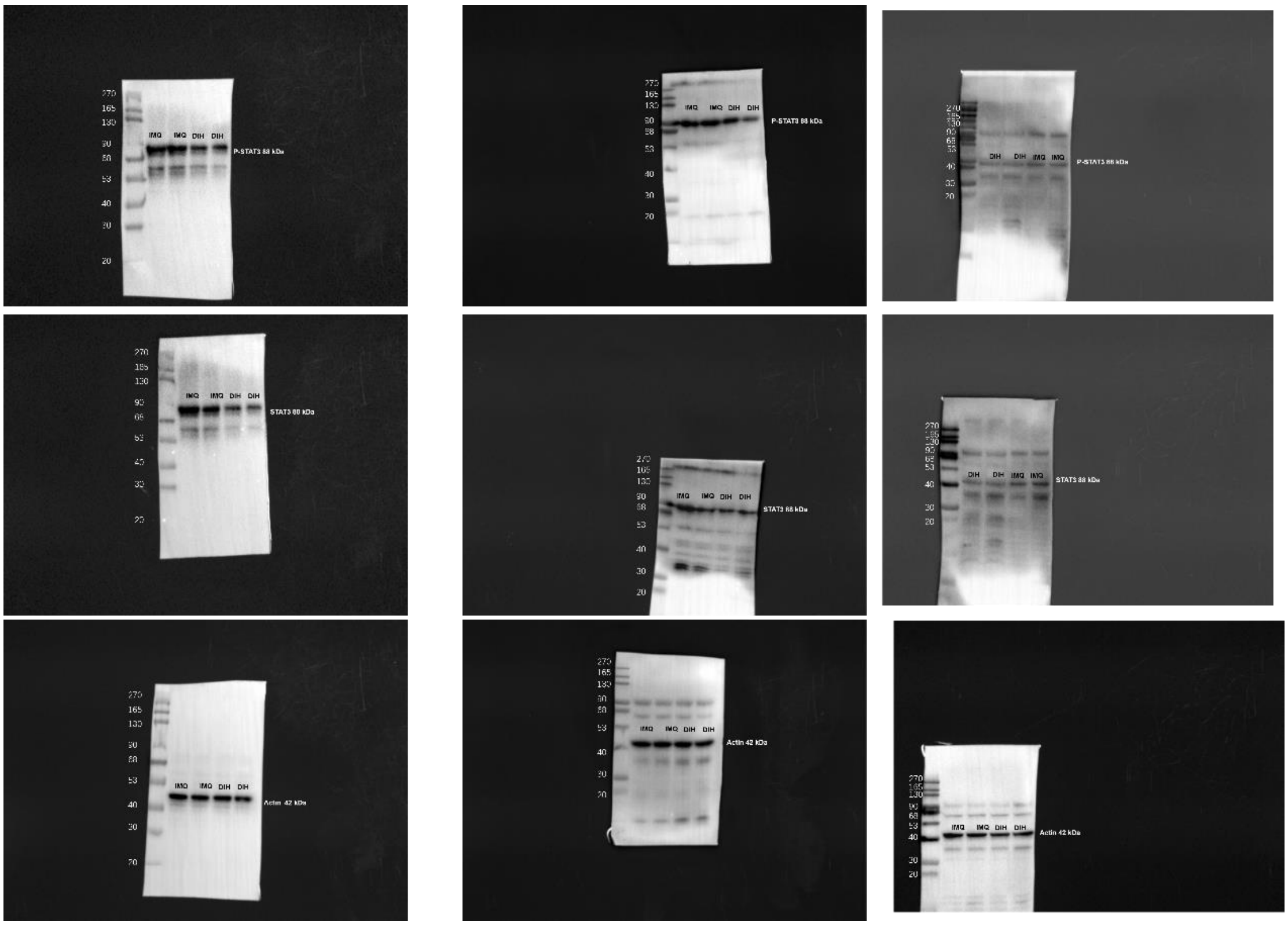

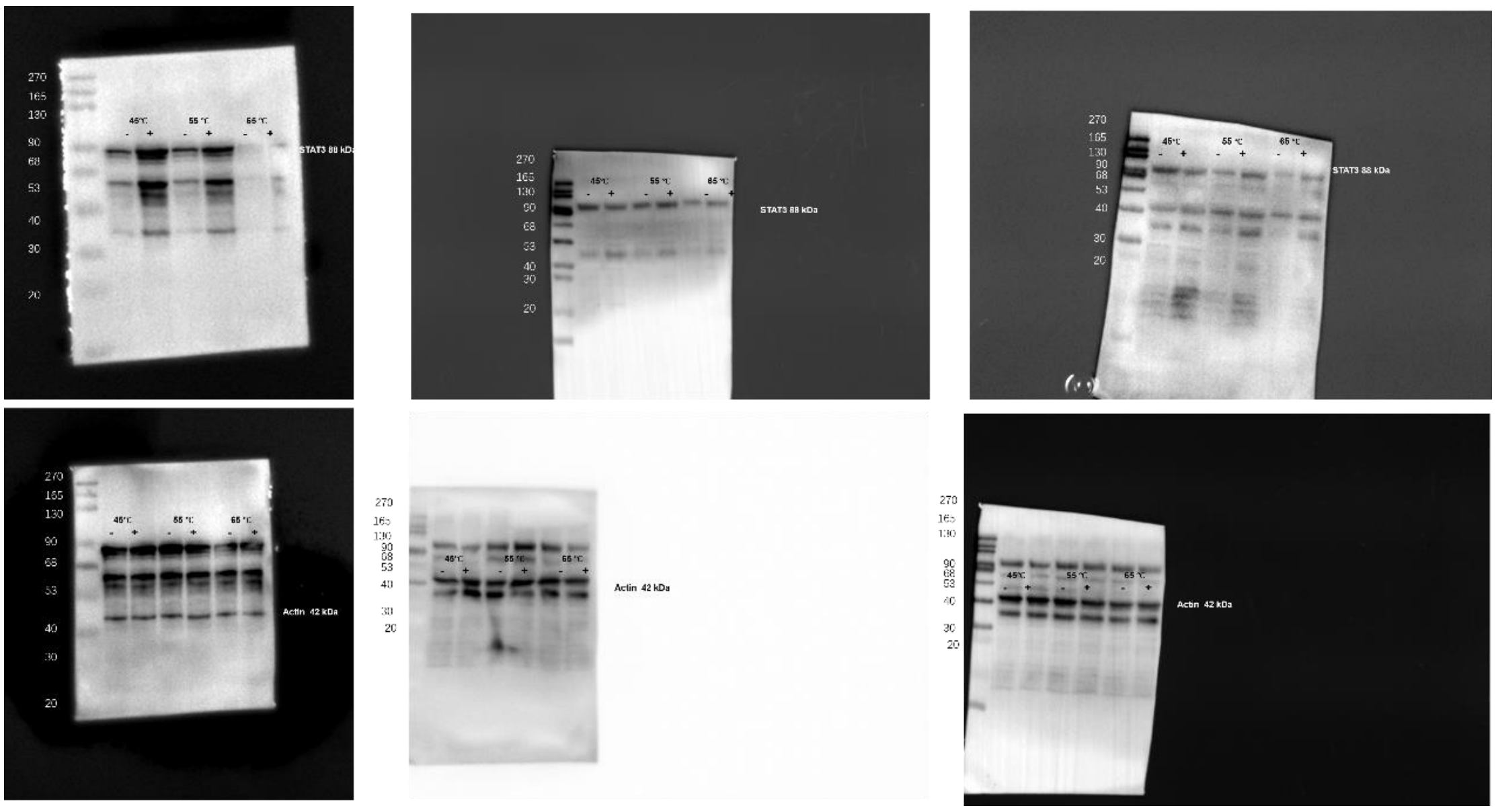

Supplement: Supplementary file 5 [file RA-016-D6RA03228A-s005.pdf]
