# Supplementary material for: Dihydrotanshinone l alleviates psoriasis-like skin lesion via suppressing STAT3 signaling and DCs-Th17 responses
Source: RSC Adv. 2026 Jul 22. Online ahead of print. doi: 10.1039/d6ra03228a (PMC13390720; doi:10.1039/d6ra03228a)
Supplement: RA-OLF-D6RA03228A-s001 [file RA-OLF-D6RA03228A-s001.pdf]

Supplementary Information (SI) for RSC Advances.
This journal is © The Royal Society of Chemistry 2026

Table S1 Primer sequence

Primer	Sequence (5'-3')
hIL-6- F	ACTCACCTCTTCAGAACGAATTG
hIL-6- R	CCATCTTTGGAAGGTTCAGGTTG
hIL-TNF-α-F	CCTCTCTCTAATCAGCCCTCTG
hIL-TNF-α-R	GAGGACCTGGGAGTAGATGAG
hIL-1β-F	AGCTACGAATCTCCGACCAC
hIL-1β- R	CGTTATCCCATGTGTCGAAGAA
hIL-23- F	GTGGGACACATGGATCTAAGAGAAG
hIL-23- R	TTTGCAAGCAGAACTGACTGTTG
hActin-F	GTACGCCAACACAGTGCTG
hActin-R	CGTCATACTCCTGCTTGCTG
mIL-1β-F	TGTGGCAGCTACCTATGTCT
mIL-1β-R	GGGAACATCACACACTAGCA
mTNF-α-F	CCTGTAGCCCACGTCGTAG
mTNF-α- R	GGGAGTAGACAAGGTACAACCC
mIL-23-F	AATAATGTGCCCCGTATCCAGT
mIL-23- R	GCTCCCCTTTGAAGATGTCAG
mIL-17A-F	TTTAACTCCCTTGGCGCAAAA
mIL-17A- R	CTTTCCCTCCGCATTGACAC
mActin-F	GGCACCACTGAACCCTAAGG
mActin-R	ACAATACCAGTTGTACGTCCAGA
