# Supplementary material for: Dihydrotanshinone l alleviates psoriasis-like skin lesion via suppressing STAT3 signaling and DCs-Th17 responses
Source: RSC Adv. 2026 Jul 22. Online ahead of print. doi: 10.1039/d6ra03228a (PMC13390720; doi:10.1039/d6ra03228a)
Supplement: RA-OLF-D6RA03228A-s002 [file RA-OLF-D6RA03228A-s002.pdf]

Supplementary Information (SI) for RSC Advances.
This journal is © The Royal Society of Chemistry 2026

Table S2 shRNA sequence

Gene name	Sequence (5'-3')
sh-STAT3-A	GCCTCATCCACATGAAC
sh-STAT3-B	GGACCTATGAGACCTTC
sh-STAT3-C	GCATGATCCAAGAAGAC
