# Supplementary material for: Dihydrotanshinone l alleviates psoriasis-like skin lesion via suppressing STAT3 signaling and DCs-Th17 responses
Source: RSC Adv. 2026 Jul 22. Online ahead of print. doi: 10.1039/d6ra03228a (PMC13390720; doi:10.1039/d6ra03228a)
Supplement: RA-OLF-D6RA03228A-s003 [file RA-OLF-D6RA03228A-s003.pdf]

Supplementary Information (SI) for RSC Advances.
This journal is © The Royal Society of Chemistry 2026

Table S3 Epidermal thickness and histological score

Group	N	Epidermal Thickness (μm)	Hyperplasia Score (0-3)	Inflammation Score (0-3)
CON	5	8.7±3.3	0.2±0.4	0.5±0.3
IMQ	5	107.2±10.2 ###	2.5±0.5###	2.2±0.4###
MTX	5	48.2±14.5**	1.3±0.2**	1.2±0.2**
DIH-L	5	36.7±10.3**	1.0±0.3**	0.9±0.2**
DIH-H	5	25.3±8.8***	0.8±0.1***	0.7±0.6***
