# Supplementary material for: Dihydrotanshinone l alleviates psoriasis-like skin lesion via suppressing STAT3 signaling and DCs-Th17 responses
Source: RSC Adv. 2026 Jul 22. Online ahead of print. doi: 10.1039/d6ra03228a (PMC13390720; doi:10.1039/d6ra03228a)
Supplement: RA-OLF-D6RA03228A-s004 [file RA-OLF-D6RA03228A-s004.pdf]

This journal is © The Royal Society of Chemistry 2026

Table S4 The 10 most significant targets of PPI network.

Number	Target name	Degree
1	EGFR	47
2	STAT3	44
3	CASP3	35
4	NFKB1	34
5	TLR4	31
6	HSP90AA1	31
7	ERBB2	30
8	KDR	26
9	MAPK14	25
10	ITGB1	22
