# Supplementary figures and images for: Dihydrotanshinone l alleviates psoriasis-like skin lesion via suppressing STAT3 signaling and DCs-Th17 responses
Source: RSC Adv. 2026 Jul 22. Online ahead of print. doi: 10.1039/d6ra03228a (PMC13390720; doi:10.1039/d6ra03228a)

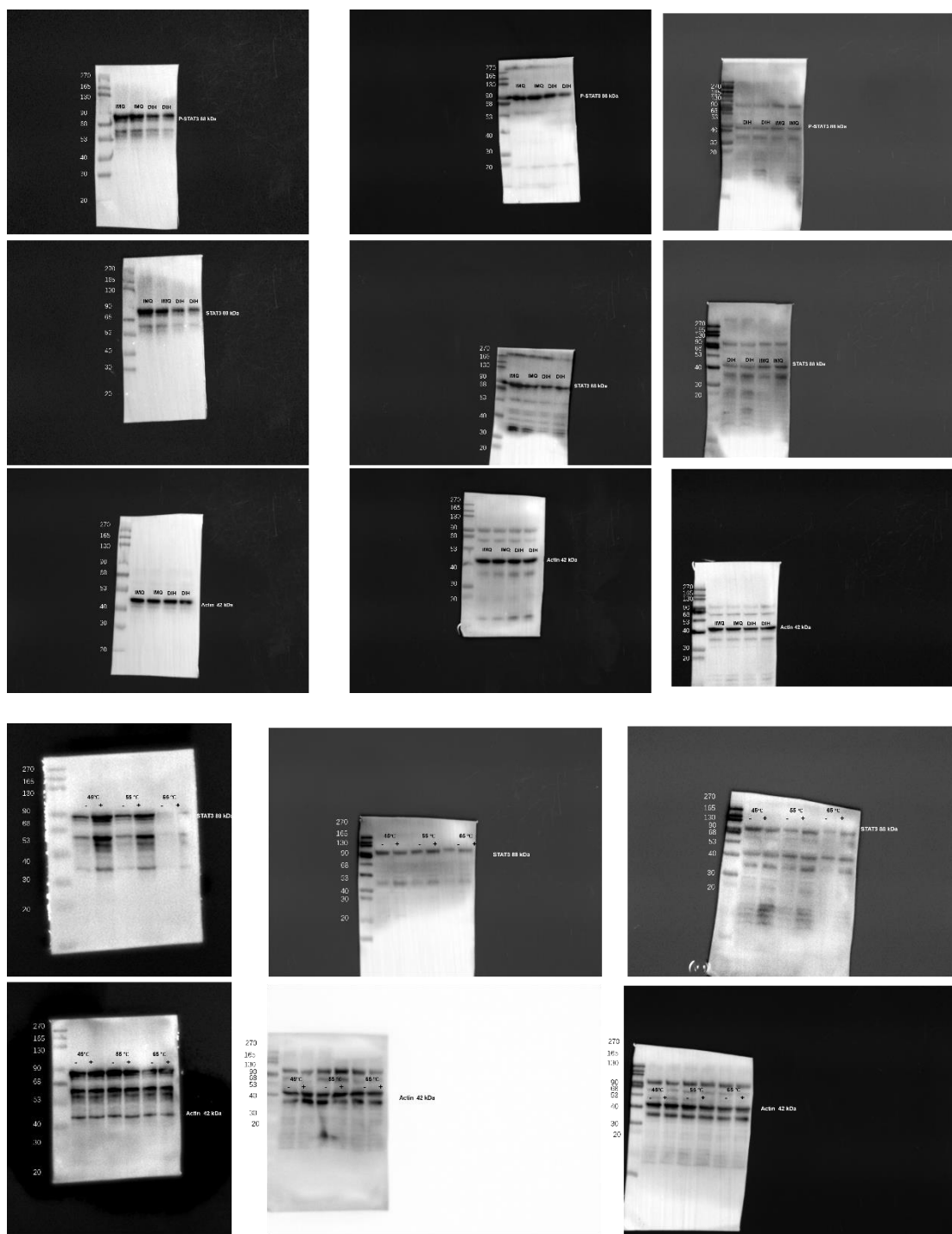

Supplement: RA-OLF-D6RA03228A-s005 [file RA-OLF-D6RA03228A-s005.pdf]
